# Supplementary figures and images for: Structural analysis of the manganese transport regulator MntR from Bacillus halodurans in apo and manganese bound forms
Source: PLoS One. 2019 Nov 18;14(11):e0224689. doi: 10.1371/journal.pone.0224689 (PMC6860424; doi:10.1371/journal.pone.0224689)

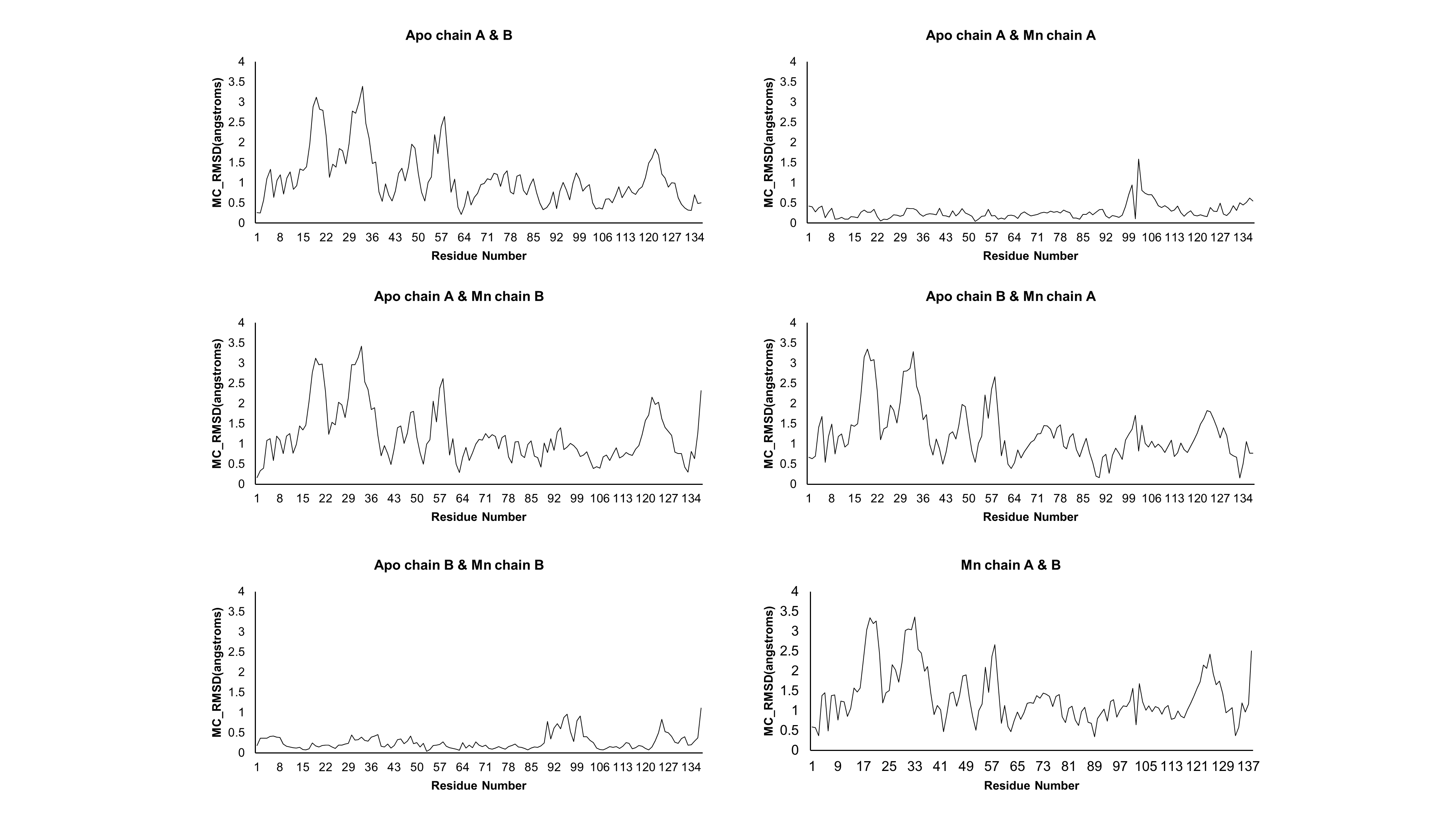

Supplement: S1 Fig — (TIF) [file pone.0224689.s001.tif]

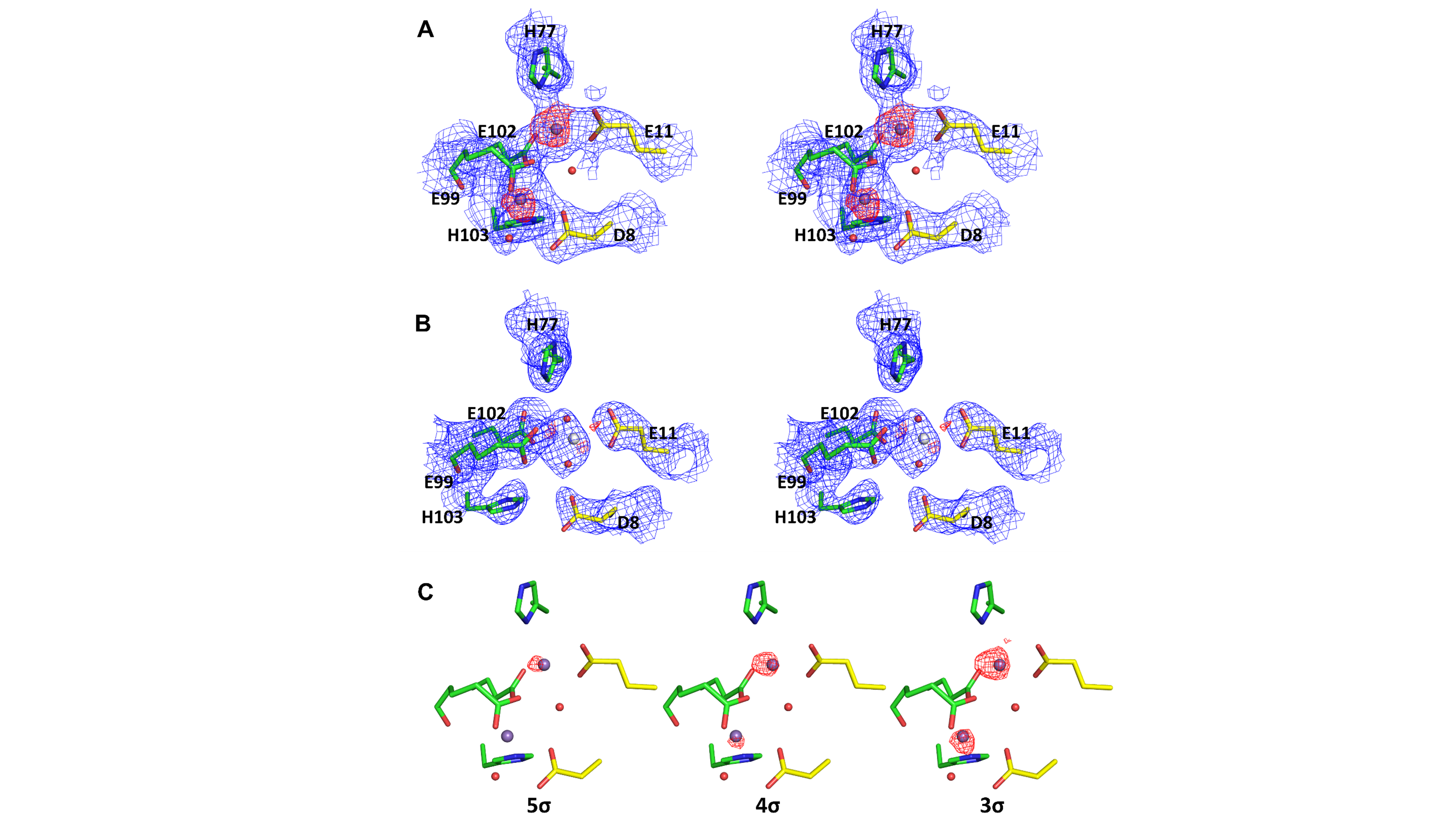

Supplement: S2 Fig — (A) Stereoview of metal binding site with binuclear manganese ions of the Mn2+-bound BhMntR. A σA-weighted electron density map (2Fo-Fc map) contoured at 1.0σ (blue). Anomalous map was calculated, contoured at 2σ (red). The Mn2+ atoms (purple) are depicted with surrounding residues (yellow sticks from domain1 and green sticks from domain2). (B) Stereoview of metal binding site with a magnesium ion in other subunit of the Mn2+-bound BhMntR. A σA-weighted electron density map (2Fo-Fc map) contoured at 1.0σ (blue). Anomalous map was calculated, contoured at 2σ (red). (C) Anomalous maps were calculated around metal binding site with binuclear manganese ions with different contour level (5σ, 4σ, and 3σ). (TIF) [file pone.0224689.s002.tif]
