# Supplementary material for: Structural analysis of the manganese transport regulator MntR from Bacillus halodurans in apo and manganese bound forms
Source: PLoS One. 2019 Nov 18;14(11):e0224689. doi: 10.1371/journal.pone.0224689 (PMC6860424; doi:10.1371/journal.pone.0224689)
Supplement: S1 Table — (DOCX) [file pone.0224689.s003.docx]

**S1 Table. Structural comparisons of *Bh*MntR with *Bs*MntR**

| r.m.s.d / Lys41 to Lys41 distance (Å) | Apo *Bh*MntR | | Mn^2+^- bound *Bh*MntR | |
| --- | --- | --- | --- | --- |
|  | Chain A | Chain B | Chain A | Chain B |
| Apo *Bs*MntR | 0.9 / 7.8 | 0.78 / 6.3 | 1.07 / 8.5 | 0.74 / 5.2 |
| Mn^2+^- bound *Bs*MntR | 0.76 / 2.4 | 0.49 / 3.4 | 0.94 / 3.3 | 0.5 / 2.8 |
| Zn^2+^- bound *Bs*MntR | 0.95 / 2.4 | 0.63 / 4.1 | 1.13 / 3.2 | 0.63 / 3.6 |
