# Supplementary material for: Structural analysis of the manganese transport regulator MntR from Bacillus halodurans in apo and manganese bound forms
Source: PLoS One. 2019 Nov 18;14(11):e0224689. doi: 10.1371/journal.pone.0224689 (PMC6860424; doi:10.1371/journal.pone.0224689)
Supplement: S5 File — (PDF) [file pone.0224689.s008.pdf]

Full wwPDB X-ray Structure Validation Report (i)

Sep 3, 2019 – 05:31 PM JST

PDB ID	:	6KTA
Title	:	Crystal structure of B. halodurans MntR in apo form
Deposited on	:	2019-08-26
Resolution	:	2.30 Å(reported)

This is a Full wwPDB X-ray Structure Validation Report.

This report is produced by the wwPDB biocuration pipeline after annotation of the structure.

We welcome your comments at *validation@mail.wwpdb.org*
A user guide is available at
https://www.wwpdb.org/validation/2017/XrayValidationReportHelp
with specific help available everywhere you see the (i) symbol.

The following versions of software and data (see references (i)) were used in the production of this report:

MolProbity	:	4.02b-467
Mogul	:	1.8.0 (224370), CSD as540be (2019)
Xtriage (Phenix)	:	1.13
EDS	:	2.4
Percentile statistics	:	20171227.v01 (using entries in the PDB archive December 27th 2017)
Refmac	:	5.8.0158
CCP4	:	7.0 (Gargrove)
Ideal geometry (proteins)	:	Engh & Huber (2001)
Ideal geometry (DNA, RNA)	:	Parkinson et al. (1996)
Validation Pipeline (wwPDB-VP)	:	2.4

CONFIDENTIAL VALIDATION REPORT

1 Overall quality at a glance (i)

The following experimental techniques were used to determine the structure:
X-RAY DIFFRACTION

The reported resolution of this entry is 2.30 Å.

Percentile scores (ranging between 0-100) for global validation metrics of the entry are shown in the following graphic. The table shows the number of entries on which the scores are based.

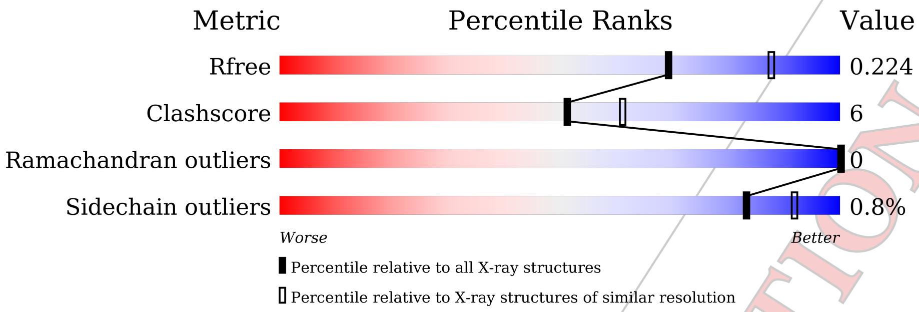

Metric	Whole archive (#Entries)	Similar resolution (#Entries, resolution range(Å))
R_{free}	111664	4477 (2.30-2.30)
Clashscore	122126	5072 (2.30-2.30)
Ramachandran outliers	120053	5022 (2.30-2.30)
Sidechain outliers	120020	5021 (2.30-2.30)

The table below summarises the geometric issues observed across the polymeric chains and their fit to the electron density. The red, orange, yellow and green segments on the lower bar indicate the fraction of residues that contain outliers for >=3, 2, 1 and 0 types of geometric quality criteria. A grey segment represents the fraction of residues that are not modelled. The numeric value for each fraction is indicated below the corresponding segment, with a dot representing fractions <=5%

Mol	Chain	Length	Quality of chain
1	A	139	86% 12% ••
1	B	139	85% 14% •

CONFIDENTIAL VALIDATION REPORT

2 Entry composition (i)

There are 3 unique types of molecules in this entry. The entry contains 2439 atoms, of which 0 are hydrogens and 0 are deuteriums.

In the tables below, the ZeroOcc column contains the number of atoms modelled with zero occupancy, the AltConf column contains the number of residues with at least one atom in alternate conformation and the Trace column contains the number of residues modelled with at most 2 atoms.

- Molecule 1 is a protein called HTH-type transcriptional regulator MntR.

Mol	Chain	Residues	Atoms	ZeroOcc	AltConf	Trace
1	A	138	Total C N O S<br>1151 733 193 221 4	0	1	0
1	B	137	Total C N O S<br>1129 717 191 217 4	0	0	0

- Molecule 2 is GLYCEROL (three-letter code: GOL) (formula: $C_3H_8O_3$).

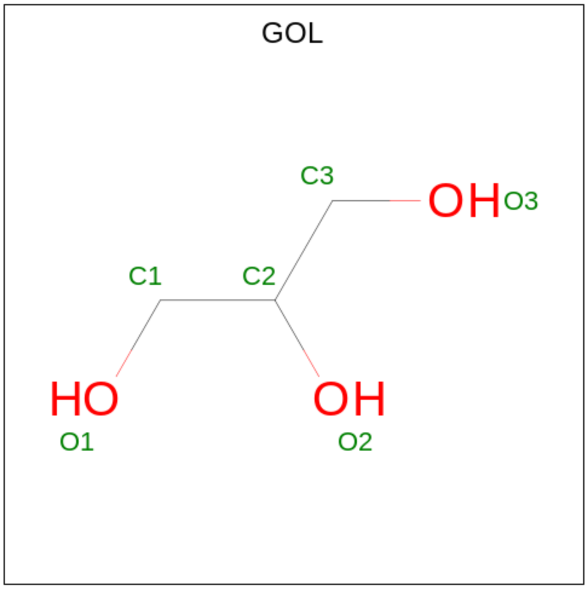

Mol	Chain	Residues	Atoms	ZeroOcc	AltConf
2	A	1	Total C O<br>6 3 3	0	0
2	A	1	Total C O<br>6 3 3	0	0
2	B	1	Total C O<br>6 3 3	0	0
2	B	1	Total C O<br>6 3 3	0	0

CONFIDENTIAL VALIDATION REPORT

- Molecule 3 is water.

Mol	Chain	Residues	Atoms	ZeroOcc	AltConf
3	A	69	Total O<br>69 69	0	0
3	B	66	Total O<br>66 66	0	0

CONFIDENTIAL VALIDATION REPORT

3 Residue-property plots (i)

These plots are drawn for all protein, RNA and DNA chains in the entry. The first graphic for a chain summarises the proportions of the various outlier classes displayed in the second graphic. The second graphic shows the sequence view annotated by issues in geometry. Residues are color-coded according to the number of geometric quality criteria for which they contain at least one outlier: green = 0, yellow = 1, orange = 2 and red = 3 or more. Stretches of 2 or more consecutive residues without any outlier are shown as a green connector. Residues present in the sample, but not in the model, are shown in grey.

- Molecule 1: HTH-type transcriptional regulator MntR

Chain A:

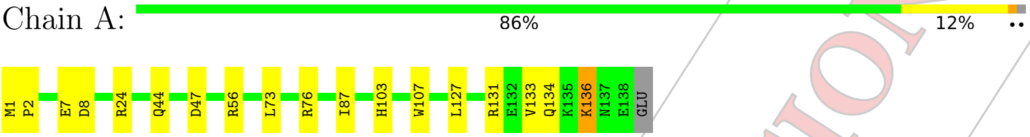

- Molecule 1: HTH-type transcriptional regulator MntR

Chain B:

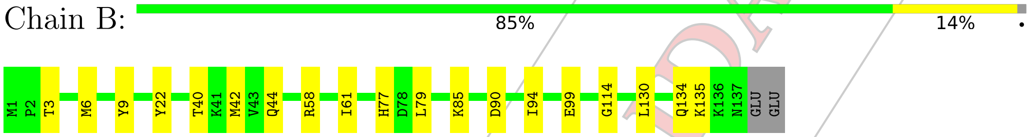

CONFIDENTIAL VALIDATION REPORT

4 Data and refinement statistics (i)

Property	Value	Source
Space group	P 21 21 21	Depositor
Cell constants<br>a, b, c, α, β, γ	39.66Å 89.20Å 109.76Å<br>90.00° 90.00° 90.00°	Depositor
Resolution (Å)	46.74 – 2.30<br>46.74 – 2.30	Depositor<br>EDS
% Data completeness<br>(in resolution range)	98.9 (46.74-2.30)<br>98.9 (46.74-2.30)	Depositor<br>EDS
R_{merge}	0.12	Depositor
R_{sym}	(Not available)	Depositor
$< I/\sigma(I) >$ [1]	4.08 (at 2.29Å)	Xtriage
Refinement program	PHENIX	Depositor
R, R_{free}	(Not available) , (Not available)<br>0.193 , 0.224	Depositor<br>DCC
R_{free} test set	876 reflections (4.89%)	wwPDB-VP
Wilson B-factor (Å^2)	30.1	Xtriage
Anisotropy	0.110	Xtriage
Bulk solvent k_{sol}(e/Å^3), B_{sol}(Å^2)	0.35 , 50.0	EDS
L-test for twinning[2]	$< \|L\| > = 0.48$, $< L^2 > = 0.32$	Xtriage
Estimated twinning fraction	No twinning to report.	Xtriage
F_o,F_c correlation	0.94	EDS
Total number of atoms	2439	wwPDB-VP
Average B, all atoms (Å^2)	35.0	wwPDB-VP

Xtriage's analysis on translational NCS is as follows: *The largest off-origin peak in the Patterson function is 9.47% of the height of the origin peak. No significant pseudotranslation is detected.*

[1] Intensities estimated from amplitudes.

[2] Theoretical values of $< |L| >$, $< L^2 >$ for acentric reflections are 0.5, 0.333 respectively for untwinned datasets, and 0.375, 0.2 for perfectly twinned datasets.

CONFIDENTIAL VALIDATION REPORT

5 Model quality (i)

5.1 Standard geometry (i)

Bond lengths and bond angles in the following residue types are not validated in this section: GOL

The Z score for a bond length (or angle) is the number of standard deviations the observed value is removed from the expected value. A bond length (or angle) with $|Z| > 5$ is considered an outlier worth inspection. RMSZ is the root-mean-square of all Z scores of the bond lengths (or angles).

Mol	Chain	Bond lengths		Bond angles	
		RMSZ	$\#|Z|>5$	RMSZ	$\#|Z|>5$
1	A	0.45	0/1176	0.58	0/1584
1	B	0.39	0/1150	0.55	0/1550
All	All	0.42	0/2326	0.56	0/3134

There are no bond length outliers.

There are no bond angle outliers.

There are no chirality outliers.

There are no planarity outliers.

5.2 Too-close contacts (i)

In the following table, the Non-H and H(model) columns list the number of non-hydrogen atoms and hydrogen atoms in the chain respectively. The H(added) column lists the number of hydrogen atoms added and optimized by MolProbity. The Clashes column lists the number of clashes within the asymmetric unit, whereas Symm-Clashes lists symmetry related clashes.

Mol	Chain	Non-H	H(model)	H(added)	Clashes	Symm-Clashes
1	A	1151	0	1146	12	0
1	B	1129	0	1120	15	0
2	A	12	0	16	1	0
2	B	12	0	16	1	0
3	A	69	0	0	2	0
3	B	66	0	0	2	0
All	All	2439	0	2298	28	0

The all-atom clashscore is defined as the number of clashes found per 1000 atoms (including hydrogen atoms). The all-atom clashscore for this structure is 6.

All (28) close contacts within the same asymmetric unit are listed below, sorted by their clash

CONFIDENTIAL VALIDATION REPORT

magnitude.

Atom-1	Atom-2	Interatomic distance (Å)	Clash overlap (Å)
1:A:44:GLN:NE2	3:A:401:HOH:O	2.06	0.89
1:B:85:LYS:HG2	1:B:94:ILE:HD13	1.77	0.67
1:A:133:VAL:HA	1:A:136:LYS:HD2	1.78	0.65
1:B:130:LEU:O	1:B:134:GLN:HG3	1.99	0.63
1:B:22:TYR:HE2	1:B:61:ILE:HD11	1.65	0.60
1:B:3:THR:HG23	1:B:6:MET:HE3	1.87	0.56
1:A:56:ARG:NH2	3:A:406:HOH:O	2.37	0.56
1:B:3:THR:OG1	1:B:6:MET:HG3	2.06	0.55
2:B:201:GOL:H11	3:B:358:HOH:O	2.10	0.52
1:A:1:MET:N	1:A:2:PRO:HD2	2.25	0.51
1:B:22:TYR:CE2	1:B:61:ILE:HD11	2.45	0.49
1:A:127:LEU:O	1:A:131:ARG:HG2	2.13	0.49
1:B:77:HIS:ND1	3:B:302:HOH:O	2.35	0.48
1:A:131:ARG:NH2	1:A:134:GLN:OE1	2.48	0.47
1:A:87:ILE:C	1:A:131:ARG:HH22	2.17	0.47
1:A:47:ASP:OD2	2:A:301:GOL:O3	2.35	0.45
1:A:24:ARG:HA	1:A:24:ARG:HD3	1.68	0.45
1:B:9:TYR:CG	1:B:42:MET:HG2	2.53	0.44
1:B:9:TYR:CD1	1:B:42:MET:HG2	2.53	0.43
1:B:85:LYS:HG2	1:B:94:ILE:CD1	2.48	0.43
1:A:8:ASP:OD1	1:A:103:HIS:NE2	2.50	0.42
1:B:79:LEU:HD11	1:B:114:GLY:HA2	2.02	0.42
1:A:7:GLU:HB3	1:A:73:LEU:HD21	2.02	0.42
1:B:135:LYS:HB2	1:B:135:LYS:HE2	1.79	0.42
1:B:22:TYR:CD2	1:B:58:ARG:HB3	2.55	0.41
1:B:90:ASP:O	1:B:94:ILE:HG13	2.20	0.41
1:A:76:ARG:HD3	1:A:107:TRP:CH2	2.55	0.41
1:B:40:THR:O	1:B:44:GLN:HG3	2.22	0.40

There are no symmetry-related clashes.

5.3 Torsion angles (i)

5.3.1 Protein backbone (i)

In the following table, the Percentiles column shows the percent Ramachandran outliers of the chain as a percentile score with respect to all X-ray entries followed by that with respect to entries of similar resolution.

The Analysed column shows the number of residues for which the backbone conformation was analysed, and the total number of residues.

CONFIDENTIAL VALIDATION REPORT

Mol	Chain	Analysed	Favoured	Allowed	Outliers	Percentiles	
1	A	137/139 (99%)	135 (98%)	2 (2%)	0	100	100
1	B	135/139 (97%)	133 (98%)	2 (2%)	0	100	100
All	All	272/278 (98%)	268 (98%)	4 (2%)	0	100	100

There are no Ramachandran outliers to report.

5.3.2 Protein sidechains (i)

In the following table, the Percentiles column shows the percent sidechain outliers of the chain as a percentile score with respect to all X-ray entries followed by that with respect to entries of similar resolution.

The Analysed column shows the number of residues for which the sidechain conformation was analysed, and the total number of residues.

Mol	Chain	Analysed	Rotameric	Outliers	Percentiles	
1	A	127/127 (100%)	126 (99%)	1 (1%)	83	92
1	B	124/127 (98%)	123 (99%)	1 (1%)	83	92
All	All	251/254 (99%)	249 (99%)	2 (1%)	83	92

All (2) residues with a non-rotameric sidechain are listed below:

Mol	Chain	Res	Type
1	A	136	LYS
1	B	99	GLU

Some sidechains can be flipped to improve hydrogen bonding and reduce clashes. All (1) such sidechains are listed below:

Mol	Chain	Res	Type
1	B	134	GLN

5.3.3 RNA (i)

There are no RNA molecules in this entry.

5.4 Non-standard residues in protein, DNA, RNA chains (i)

There are no non-standard protein/DNA/RNA residues in this entry.

CONFIDENTIAL VALIDATION REPORT

5.5 Carbohydrates (i)

There are no carbohydrates in this entry.

5.6 Ligand geometry (i)

4 ligands are modelled in this entry.

In the following table, the Counts columns list the number of bonds (or angles) for which Mogul statistics could be retrieved, the number of bonds (or angles) that are observed in the model and the number of bonds (or angles) that are defined in the Chemical Component Dictionary. The Link column lists molecule types, if any, to which the group is linked. The Z score for a bond length (or angle) is the number of standard deviations the observed value is removed from the expected value. A bond length (or angle) with $|Z| > 2$ is considered an outlier worth inspection. RMSZ is the root-mean-square of all Z scores of the bond lengths (or angles).

Mol	Type	Chain	Res	Link	Bond lengths			Bond angles		
					Counts	RMSZ	$\#\|Z\| > 2$	Counts	RMSZ	$\#\|Z\| > 2$
2	GOL	A	301	-	5,5,5	0.27	0	5,5,5	0.76	0
2	GOL	A	302	-	5,5,5	0.30	0	5,5,5	0.54	0
2	GOL	B	201	-	5,5,5	0.35	0	5,5,5	1.02	0
2	GOL	B	202	-	5,5,5	0.34	0	5,5,5	0.34	0

In the following table, the Chirals column lists the number of chiral outliers, the number of chiral centers analysed, the number of these observed in the model and the number defined in the Chemical Component Dictionary. Similar counts are reported in the Torsion and Rings columns. '-' means no outliers of that kind were identified.

Mol	Type	Chain	Res	Link	Chirals	Torsions	Rings
2	GOL	A	301	-	-	4/4/4/4	-
2	GOL	A	302	-	-	0/4/4/4	-
2	GOL	B	201	-	-	3/4/4/4	-
2	GOL	B	202	-	-	4/4/4/4	-

There are no bond length outliers.

There are no bond angle outliers.

There are no chirality outliers.

All (11) torsion outliers are listed below:

Mol	Chain	Res	Type	Atoms
2	A	301	GOL	C1-C2-C3-O3
2	B	202	GOL	O1-C1-C2-O2

Continued on next page...

CONFIDENTIAL VALIDATION REPORT

Continued from previous page...

Mol	Chain	Res	Type	Atoms
2	A	301	GOL	O1-C1-C2-O2
2	B	202	GOL	O1-C1-C2-C3
2	B	201	GOL	O1-C1-C2-C3
2	A	301	GOL	O1-C1-C2-C3
2	B	201	GOL	C1-C2-C3-O3
2	B	201	GOL	O2-C2-C3-O3
2	A	301	GOL	O2-C2-C3-O3
2	B	202	GOL	C1-C2-C3-O3
2	B	202	GOL	O2-C2-C3-O3

There are no ring outliers.

2 monomers are involved in 2 short contacts:

Mol	Chain	Res	Type	Clashes	Symm-Clashes
2	A	301	GOL	1	0
2	B	201	GOL	1	0

5.7 Other polymers (i)

There are no such residues in this entry.

5.8 Polymer linkage issues (i)

There are no chain breaks in this entry.

CONFIDENTIAL VALIDATION REPORT

6 Fit of model and data (i)

6.1 Protein, DNA and RNA chains (i)

Unable to reproduce the depositors R factor - this section is therefore empty.

6.2 Non-standard residues in protein, DNA, RNA chains (i)

Unable to reproduce the depositors R factor - this section is therefore empty.

6.3 Carbohydrates (i)

Unable to reproduce the depositors R factor - this section is therefore empty.

6.4 Ligands (i)

Unable to reproduce the depositors R factor - this section is therefore empty.

6.5 Other polymers (i)

Unable to reproduce the depositors R factor - this section is therefore empty.

CONFIDENTIAL VALIDATION REPORT
