# Supplementary material for: Structural analysis of the manganese transport regulator MntR from Bacillus halodurans in apo and manganese bound forms
Source: PLoS One. 2019 Nov 18;14(11):e0224689. doi: 10.1371/journal.pone.0224689 (PMC6860424; doi:10.1371/journal.pone.0224689)
Supplement: Attachment — Submitted filename: rebuttal_letter.docx [file pone.0224689.s010.docx]

Date: Oct. 18, 2019

Dear Eugene A. Permyakov, Ph.D. Academic Editor, PLOS ONE

Reference code PONE-D-19-24432

TITLE: “**Structural analysis of the manganese transport regulator MntR from Bacillus halodurans in apo and manganese bound forms**”

Thank you very much for your kindly informing me of the reviewer’s comments on my manuscript submitted for a possible publication in the *PLOS ONE*. Please find the uploaded revised manuscript at the web address.

The revised manuscript takes into account all the comments made by the reviewers and editor. A separate list is attached to this letter to describe all the changes in detail.

Sincerely yours,

Jae Young Lee

Department of Life Science, Dongguk University-Seoul, 32, Dongguk-ro, Ilsandong-gu, Goyang-si, Gyeonggi-do 10326, Korea

Tel: 82-31-961-5136; Fax: 82-31-961-5108

E-mail: jylee001@dongguk.edu

Encl.

Journal Requirements:

1. << When submitting your revision, we need you to address these additional requirements.

Please ensure that your manuscript meets PLOS ONE's style requirements, including those for file naming. The PLOS ONE style templates can be found at

http://www.journals.plos.org/plosone/s/file?id=wjVg/PLOSOne_formatting_sample_main_body.pdf and http://www.journals.plos.org/plosone/s/file?id=ba62/PLOSOne_formatting_sample_title_authors_affiliations.pdf >>. Our revised manuscript was generated with PLOS ONE style templates as requested.

2. << We found text overlap between the current submission and your previously published works outside the Method section:

- https://www.nature.com/articles/s41598-018-31676-z

- http://scripts.iucr.org/cgi-bin/paper?S1399004714004118

- http://koreascience.or.kr/article/JAKO201425560113942.page

In your revision ensure you cite all your sources (including your own works), and quote or rephrase any duplicated text outside the methods section. Further consideration is dependent on these concerns being addressed." >> - We carefully checked text overlaps between the current submission and your previously published works outside the Method section. Main overlaps were found in Acknowledgement Section and the other minor overlap was rephrased on lines 291-293, page 13, stating that “Although the functional assignment of metal binding site for *Bh*MntR is tentative, this structural model is applicable to other MntR homologous structures.”

3. << We note that you have stated that you will provide repository information for your data at acceptance. Should your manuscript be accepted for publication, we will hold it until you provide the relevant accession numbers or DOIs necessary to access your data. If you wish to make changes to your Data Availability statement, please describe these changes in your cover letter and we will update your Data Availability statement to reflect the information you provide. >> - We already provided the accession numbers (6KTA and 6KTB) for PDB coordinates and structure factors and those data were included in Supporting Information.

List of changes by reviewer 1

1. << Important concern: Nothing is said by the authors about anomalous scattering. They might have generated stronger anomalous signal from the Mn had they collected data at a longer x-ray wavelength, and in view of this it’s not clear why the wavelength was chosen to be at 0.979 Ang (which is a good choice for selenium studies, not applicable here). Maybe the authors could get lucky and anomalous signal in a map would be detectable even at the sub-optimal wavelength where they collected the data (f’’ for Mn looks to be about 1.3 electrons at 0.979 Ang). The authors should calculate an anomalous difference map to see if the binuclear site shows up. If it does, then they could go on to look at the other, supposedly unoccupied, site. There they inferred a Mg ion, but as noted above it doesn’t make a lot of sense that a Mg would be there when it wasn’t there in the apo form. It’s conceivable that the density in the other site is a partially occupied Mn (not Mg). And if so maybe an anomalous map would give an indication. Having a better assignment of what’s in the other site would improve the findings of the paper. >> - We calculated an anomalous difference map with Mn^2+^-bound *Bh*MntR data and found the two clear peaks showing the manganese binuclear ions in metal binding site. The additional information was added on lines 200-202, page 10, stating that “We obtained Mn^2+^-bound crystals by soaking with 50 mM MnCl_2_ in apo crystals, and confirmed using an omit map and an anomalous map showing two peaks at the counter levels even at 5σ (Fig 2A and S2 Fig).”. In addition, the requested anomalous maps of Mn^2+^-bound *Bh*MntR structures was added to supplemental figure (S2 Fig).

2. << There are some places where an editing for English usage will be important (e.g. recombination vs recombinant). >> - The English spelling was carefully checked and corrected on line 104, page 5, stating that “recombinant”.

3. << Lines 61-69 in the Introduction discuss some structural details of the MntR protein. As it is written is sounds like the authors might be describing results from the present study (which would be out of place in the Introduction), whereas they are presumably discussing what is known about the structure from prior work. This should be made clearer, e.g. “From previous structural studies it is understood that…” >> - The requested information was added as recommended on lines 62-69, page 3, stating that “From previous structural studies it is known that the metal binding site of *B. subtilis* MntR consists of several residues including Asp8 and Glu11 in domain 1, and His77, Glu99, Glu102 and His103 in domain 2.”

4. << The R-values are reported with a number of significant digits that seems one too many. >> - The R-values were corrected as recommended in text and table.

5. << The legend to Fig 2 needs to explain better the distinction (in panels b AND c) between the site where the binuclear site is seen vs the manganese site. >> - The original Fig2 legend sentence was changed on lines 212-217, page 10, stating that “(B) Metal binding site with binuclear manganese ions. The coordination with binuclear manganese ions and the distance between Mn_c_ and the backbone carbonyl oxygen of Glu99 are shown in yellow and red, respectively. (C) Metal binding site with a magnesium ion. Unlike binuclear manganese ions binding, the magnesium ion (gray) forms a mononuclear cluster and the His77 made a hydrogen bond with Glu81 via a water (red).”

6. << The authors show 2Fo-Fc and omit (difference) maps to show the metal sites. This is ok. But they have the opportunity here to also calculate and show maps that are based on differences between the Fobs from the two data sets (i.e. Fobs(+metal) – Fobs(-metal), phased with a model without metals). This kind of map is a more direct examination of the observed differences and less model-dependent. The authors should examine this kind of map. Presumably it will show up the binuclear Mn cluster just as the Fo-Fc map did. But the authors might discern more about the unexpected magnesium site. Currently they say that no magnesium is seen in the ‘apo’ structure, despite the high concentration of Mg in the crystallization conditions. If Mg is really absent from that site in the apo form, then they should see a positive peak there in the Fo(+metal) – Fo(-metal) map.>> - We also calculated the Fo(+metal) – Fo(-metal) map. The two positive peaks were clearly shown around metal binding site indicating the binuclear manganese ions but no clear map was shown in magnesium binding site in the other subunit. In addition, the anomalous maps were calculated and shown in Supporting Information (S2 Fig).

List of changes by reviewer 2

1. << This manuscript describes MntR (from Bacillus halodurans) structures in the apo and metal bound states. The assignment of Mn2+ and Mg2+ in the binding sites was not solid. It needs to be substantialized by additional measurements such as X-ray anomalous signals. The reported structures do not provide new biological insights in addition to the many existing MntR structures. >> - We calculated an anomalous difference map with Mn^2+^-bound *Bh*MntR data and found the two clear peaks showing the binuclear manganese ions in metal binding site. The additional information was added on lines 200-202, page 10, stating that “We obtained Mn^2+^-bound crystals by soaking with 50 mM MnCl_2_ in apo crystals, and confirmed using an omit map and an anomalous map showing two peaks at the counter levels even at 5σ (Fig 2A and S2 Fig).”. In addition, the requested anomalous maps of *Bh*MntR structures was added to Supplementary figure (S2 Fig).
